# Diffuse myometrium thinning and placenta accreta spectrum in a patient with systemic lupus erythematosus (SLE): a case report and review of the literature

**DOI:** 10.1186/s12884-022-04864-z

**Published:** 2022-07-02

**Authors:** Tomohiro Mitoma, Kei Hayata, Satomi Yokohata, Akiko Ohira, Chiaki Kashino, Satoe Kirino, Kazumasa Tani, Jota Maki, Eriko Eto, Hisashi Masuyama

**Affiliations:** Department of Obstetrics and Gynecology, Graduate School of Medicine Dentistry and Pharmaceutical Sciences, Department of Obstetrics and Gynecology, Okayama University, 2-5-1 Shikata-cho, Kita-ku, Okayama city, Okayama, 700-8558 Japan

**Keywords:** Lupus, Myometrium, Placenta accreta spectrum, Estrogen, Uterine atony

## Abstract

**Background:**

Cases of uterine wall thinning and placental abnormalities complicated with systemic lupus erythematosus (SLE) during pregnancy have been reported in Asian countries for ten years. Long-term steroid use can cause muscle degeneration, but the mechanism of myometrium thinning was not known. Through the review of published articles, this report is the first review of cases to discuss the pathogenesis and clinical features of thinned myometrium and placenta accreta spectrum (PAS) in pregnant patients with SLE.

**Case presentation:**

A twenty-nine-year-old primigravida with a history of lupus enteritis and paralytic ileus had a natural conception after less than two years of steroid treatment. An ultrasonographic study showed a thin uterine wall with a widespread thick placenta on the entire surface of the uterine cavity in the third trimester. At the 39th gestational week, she underwent a cesarean section due to the failure of the uterus to contract, even though the injection of oxytocin. There were several engorged vessels on the surface of the anterior uterine wall at the time of laparotomy. We decided to perform a hysterectomy because diffuse PAS replaced her uterus.

**Conclusion:**

A review of reported cases and our case shows an unusual complication of SLE that might be related to the particular condition of the estrogen-mediated immune system. Clinicians should always pay attention to the possibility of uterine wall thinning as uterine atony and the structural abnormality of the placenta for SLE patients with the unscarred uterus.

**Supplementary Information:**

The online version contains supplementary material available at 10.1186/s12884-022-04864-z.

## Background

Systemic lupus erythematosus (SLE) is a systemic autoimmune disease that is relatively common in reproductive-age women. SLE in pregnancy induces various obstetric complications, such as miscarriage, premature delivery, and preeclampsia [1]. There have been reported placenta accreta spectrum (PAS) cases in pregnant women with SLE over the last ten years [2–7]. These cases were complicated with uterine wall thinning, which led to a rupture of the uterus and hysterectomies. The relationship between SLE and uterine wall thinning is unknown, so it is difficult to predict before the labor. Herein, we report a case of entire uterine wall thinning resulting in diffuse PAS that occurred in the unscarred uterus of a woman with SLE.

## Case presentation

**Fig. 1:**
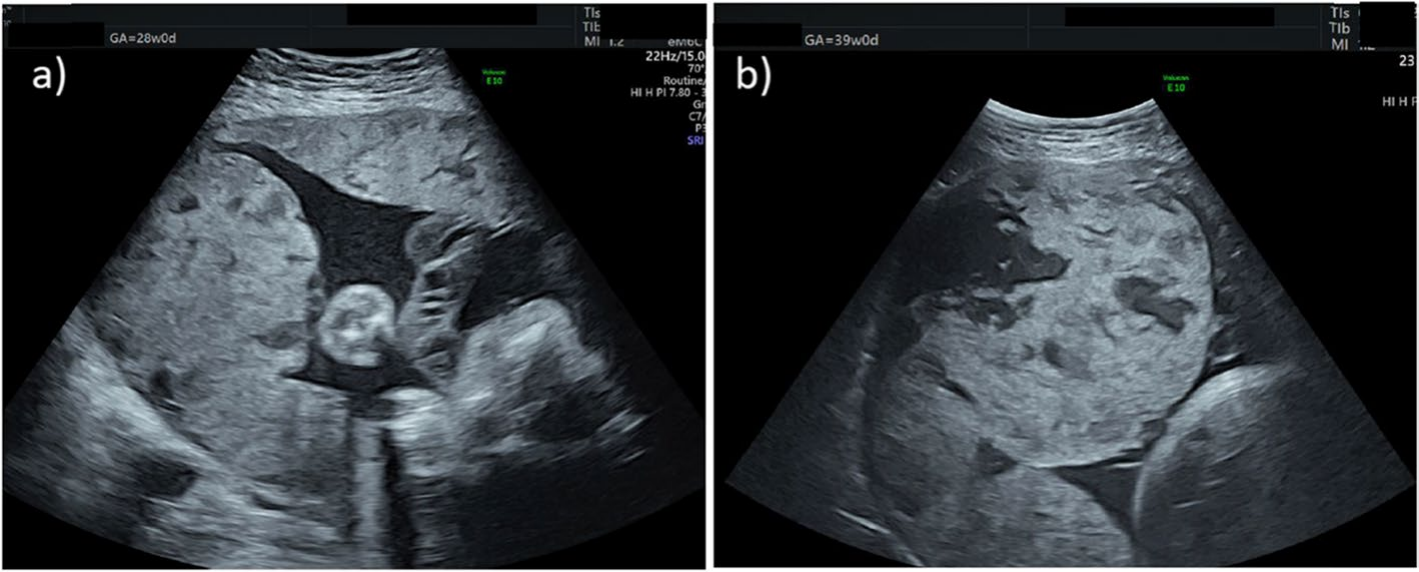
Ultrasonography image. **a** the placenta was at anterior to bottom of the uterus without clear borderline of the placenta and the muscle layer at the 28th gestational week. **b** Thickened placenta with large lacunae at the bottom of the uterus at 39th gestational week

A 29-year-old primigravida with SLE history began to visit our obstetric hospital for perinatal checkups. She was diagnosed with SLE at 26 after an episode of repeated paralytic ileus one year prior. Her SLE was treated with prednisolone and mycophenolate mofetil for 1 year and 9 months, followed by hydroxychloroquine. The immunosuppressant was shifted to tacrolimus (3 mg/day) 6 months before the conception. Her disease activity was stable enough to continue her pregnancy without any dose adjustment of tacrolimus. She did not have any history of surgery on the uterus or infertility treatment. Her pregnancy almost had a standard course, except she had the shortening of the cervix of 15 mm in length without uterine contractions in her second trimester. Ultrasound findings of the uterus showed posterior wall thickness was only 2 mm and fluid accumulation in the Douglas pouch simultaneously. In addition, her cervical length was shortened to 8 mm in the early stage of the third trimester, but she did not complain of any uterine contractions. We considered it was cervical incompetency and observed it closely on bed rest at home. The placenta was on the anterior wall to the bottom of the uterus in the second trimester, but gradual spreading to the entire surface was confirmed with ultrasonography in the third trimester (Fig. 1). Her fetus’s growth had a standard course. There was no sign of hypertensive disorder during pregnancy. We could not notice the structure relationship between the placenta and the thin uterine wall since no major pregnancy complications related to SLE were not remarkable during regular checkups.

**Fig. 2:**
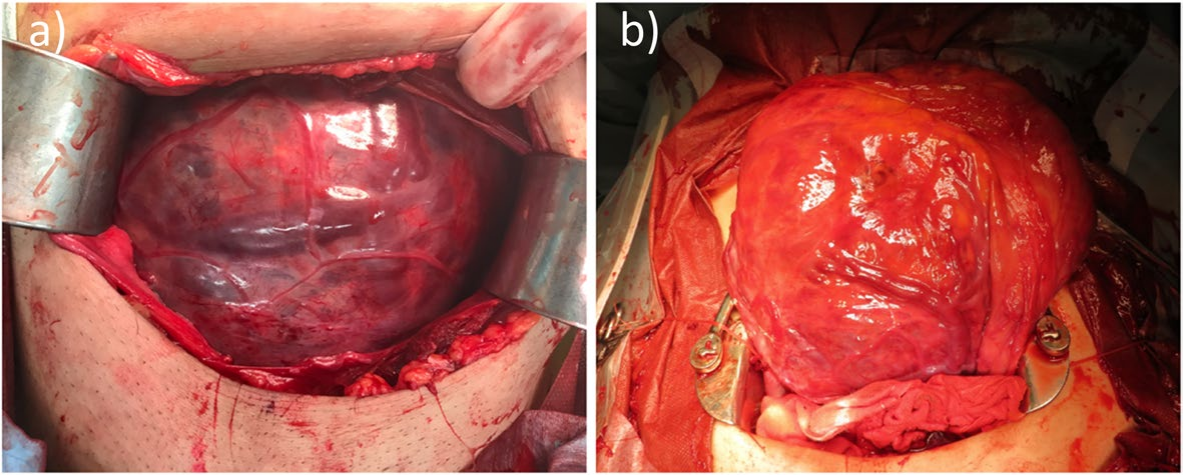
Photo image. **a** Anterior surface of the uterus with engorged vessels at the time of laparotomy. **b** Atonic uterus lifted out of the pelvic cavity after bleeding control

At 39 weeks of gestation, she was admitted due to a rupture of the membrane. Her cervix was dilated to 3 cm with 80% effacement on admission. Her white blood cell count was 13.5 × 106/ml, neutrophils were 90%, and C-reactive protein was 8.0 mg/dL. Her body temperature was 37.8 degrees Celsius (oC). Other blood tests and vital signs were unremarkable. We started antibiotic injections and induced labor with oxytocin at admission due to a low-grade fever. Although the injection dose of oxytocin rose to 20 mU/min, midwives reported softening her abdomen, and uterine contractions were did not occur at all. An emergency cesarean section (CS) was performed due to failure of the uterus to contract, and intrauterine infection was suspected. During the CS, there were several engorged vessels on the surface of the anterior uterine wall at the time of laparotomy (Fig. 2a). We confirmed that this engorged vessel was a part of the bulging placenta with intraoperative ultrasonography. By the examination through color ultrasonography, we found a placenta-free spot at the lower edge of the placenta. A healthy male baby (2908 g) with an Apgar score of 8/9(1 min/5 min) was delivered. The uterus did not contract at all even though we intramuscularly injected the 5 IU/ml of oxytocin (Fig. 2b). We closely observed the placement of the uterus and placenta. The uterine wall was fragile, and the placenta obscured her entire uterus. We decided to perform a hysterectomy because diffuse PAS replaced her uterus. The amount of blood loss was 5400 ml in total. She stayed in the intensive care unit for two days. The condition after CS and hysterectomy was good, and the patient and baby were discharged on the sixth day postoperatively.

**Fig. 3:**
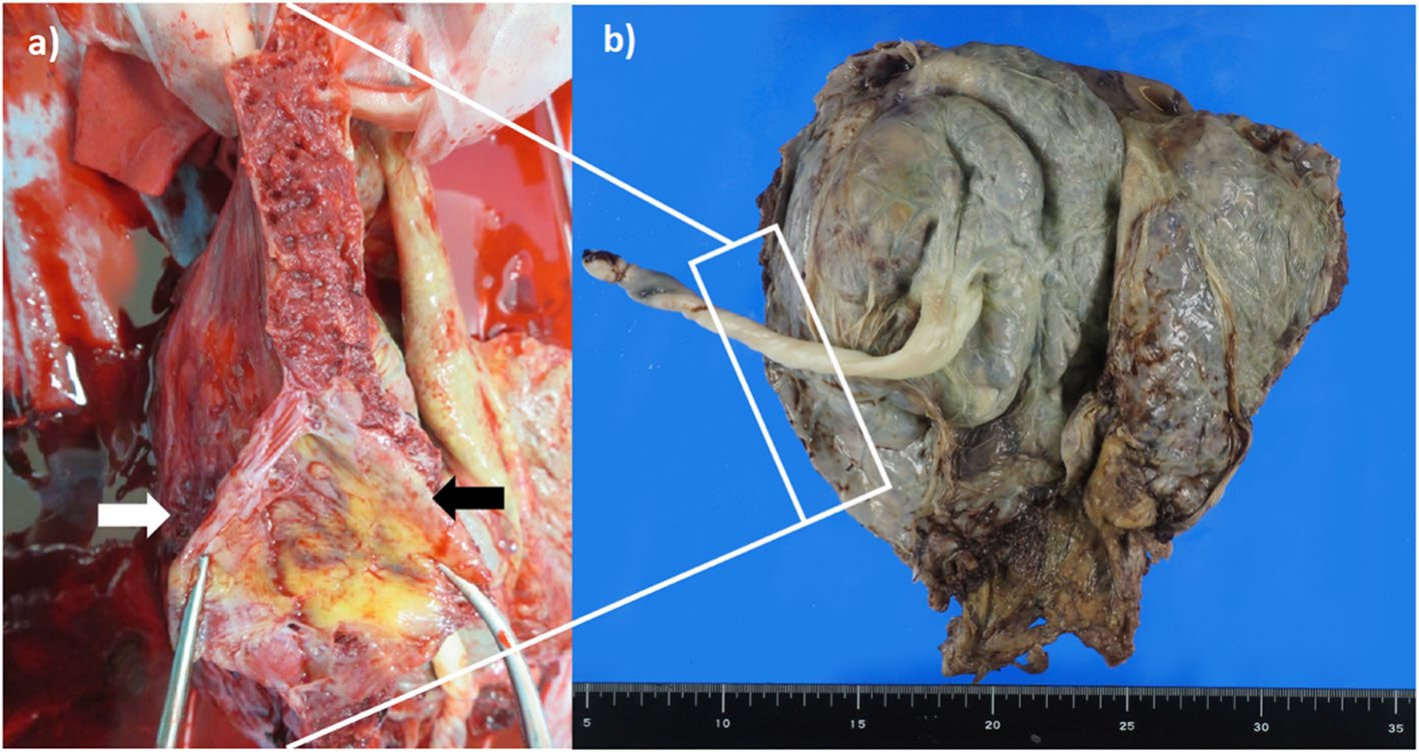
**a** Longitudinal image of the uterus and placenta after hysterectomy. Placenta (black arrow) attached to extreme thinned uterine wall (white arrow). **b** Gross photography of the uterus, which incised the anterior wall, and the placenta was attached to the widespread entire uterine wall

Gross examination of the uterus revealed the uterine wall thinning with diffuse placental attachment (Fig. 3a, b). Histopathological study showed that the thickness of the myometrium layer was less than 1 mm at not only the attachment site of the placenta but also most parts of the uterus (Figure e in supplementary information). Perivillous fibrin deposition was also observed at the placenta-invaded site of the myometrium (Figure b in supplementary information). We confirmed the diagnosis of PAS by an appreciation of part of villus component that reached the serous membrane of the uterus. Fibrosis was seen within the layer of myometrium through the trichrome staining (Figure c in supplementary information). Inflammatory cell infiltration in vessels was visible at the site of placenta-free normal myometrium through hematoxylin and eosin (HE) staining (Figure d in supplementary information).

## Discussion and conclusion

The relationship between SLE during pregnancy and abnormal myometrium with PAS has been reported in Asian countries for ten years [2–7]. There were 12 reports (7 English and 5 Japanese articles), and 14 patients were identified, including our patient. Interestingly, all patients were from Asian counties (Japan [3, 4, 7]), South Korea [2, 6] Vietnam [5]. Here, we reviewed eight patients from 6 English articles, including our patients with SLE during pregnancy with uterine wall thinning and PAS (Table 1). The mean maternal age was 33 years (range, 29–36 years). All patients had to undergo CS to deliver the babies. Two patients had a rupture of the uterus. All patients failed to have uterine preservation, leading to hysterectomies at that time of the CS. 5 out of 8 patients had a risk of PAS, such as in vitro fertilization [8], placenta previa, and a history of uterine curettage [9]. 6 out of 8 patients had a history of steroid use for more than five years and used them during pregnancy. Our case was different from this case series in which the duration of corticosteroid use was shorter than two years. Corticosteroids administration was ceased six months before conception or during pregnancy.

**Table 1:** Case reports of SLE during pregnancy with uterine wall thinning and PAS

Author,year	Age(y)	Parity	PSL duration(y)	PSL use during pregnancy	Risk factors of PAS	Obstetric complication	GA at labror(wk)	Induction labor	Delivery	Treatment	Amount of blood loss(ml)
Noh JJ et al., 2013 [2]	36	0	19	Yes	IVF	Rupture uterus	23	None	CS	Hysterectomy	NS
Tokushige Y et al., 2017 [3]	35	0	5	Yes	IVF	GH	39	Yes (Oxytocin)	CS	Hysterectomy	4500
Tomimatsu T et al., 2021 [4]	32	0	8	Yes	IVF	PE	35	Yes (Oxytocin)	CS	Hysterectomy	2200
Mittal N et al., 2018 [5]	36	2	9	Yes	Placenta previa	Placenta previa	36	None	CS	Hysterectomy	6000
Kim HM et al., 2020 [6]	33	0	10	Yes	None	Rupture uterus	34	None	CS	Hysterectomy	NS
Kim HM et al., 2020 [6]	30	0	8	NS	None	None	38	None	CS	Hysterectomy	NS
Inoue A et al., 2020 [7]	37	0	19	Yes	IVF	NRFS,FGR	36	None	CS	Hysterectomy	5860
Our case	29	0	2	none	none	none	39	Yes (Oxytocin)	CS	Hysterectomy	5400

Abbreviations: *PSL* predonisolone;GA,gestational age, *IVF* in vitro fertilization, *CS* cesarean section, *GH* gestational hypertension, *PE* preeclampsia, *NRFS* non-reassuring fetal status, *FGR* fetal growth restriction, *NS* not stated, *SLE* systemic lupus erythematosus, *PAS* placenta accrete spectrum

It has been thought that prolonged steroid use could cause the impairment of protein synthesis [10] and result in the thinning of the myometrium. However, the histopathology of patients in this case review did not show any feature of corticosteroid-induced myopathies, such as atrophy with variability in fiber size and centralization of nuclei [11]. Moreover, although women of childbearing age with other autoimmune diseases such as rheumatoid arthritis often use steroids in the long term, no case of diffused PAS other than with SLE has been reported in our search. By reviewing published articles on the length of steroid treatment and medical history, the pathophysiological feature of the antiestrogenic effect of SLE might lead to a modified structure of the uterine wall. Estrogen has a vital role in inducing hypertrophy of the myometrium and endothelial proliferation during normal pregnancies [12]. Several studies indicated lower estrogen levels in pregnant patients with SLE than in patients with normal pregnancies throughout pregnancy [13, 14]. When low estrogen levels disturb hypertrophy of myometrium and endothelial proliferation in SLE patients, it can lead to uterine wall thinning. Our hypothesis has a limitation: the lack of data of hormonal assessment during pregnancy in this case.

In this case, the histological study revealed multiple invasion sites of the chorionic plate into the myometrium. We could also appreciate a microscopical observation of fibrosis and vasculitis at the myometrium. Interstitial fibrosis to myocardial involvement [15] and gynecological vasculitis in SLE have been reported to date [16], causing activating the complement cascade to damage the microvascular structure of the uterus. In particular, our patient had repeated paralytic ileus and lupus enteritis without steroid treatment for one year. The non-treatment period of one year might have caused damage to endometrial alignment and myometrium before conception. Considering the histological features of multiple placental invasion and vasculitis, we thought chronic inflammation might have provoked the damage to the endometrium and myometrium, which cause multiple placental invasions into the thinned myometrium. These histological features of vasculitis were not investigated in other cases, and more cases are needed to support this pathological characteristic.

Clinical signs of uterine atony and ultrasonographic features in this case report are essential from a clinical perspective. The clinical signs of uterine atony during induction labor with oxytocin injection can lead to suspicion of abnormal uterus wall structure in SLE patients. In the case series, three patients had oxytocin administration during induced labor. These cases experienced continuous uterine atony after the baby’s birth, even though intramuscular injection of the uterotonic agent. Tomimatsu et al. [4]. expressed the atonic situation as a collapsed uterus. Uterine atony and PAS can result in massive postpartum bleeding (mean amount of 4800 ml), which was the reason to perform hysterectomies in most cases. In our case, there were clinical signs that indicated abnormalities in the contractive function of the uterine muscle from the second trimester. First, the patient had cervical length shortening without uterine contraction in her second trimester. Second, the uterine fundus was softened like a marshmallow when midwives examined the induction of labor. Uterine atony might be a pivotal notification to lead further investigation and prepare for organizing a multidisciplinary team before delivery.

To diagnose the PAS before labor is always challenging in patients with an unscarred uterus [17]. We should note the structural relationship of placental invasion to the uterine wall by ultrasonography in SLE patients from the beginning of pregnancy. Inoue et al. [7]. reported placenta thickening and missing decidual blood flow detected by superb microvascular imaging of ultrasonography that led to a diagnosis of PAS before labor. Mittal et al. [5]. reported that the loss of myometrial definition in uterine with increased placental lacunae vascularity was the critical finding to suspect PAS. In our patient, there was a thick placenta with an increased size of placental lacunae at first, and then it spread throughout the uterus in the third trimester.

In conclusion, we have presented an unusual complication of SLE that might be related to the unique condition of the estrogen-mediated immune system. Clinicians should always pay attention to the possibility of uterine wall thinning as uterine atony and the structural abnormality between the placenta and uterine wall for SLE patients without any risk.

## Supplementary Information

**Additional file 1:** **Figure **a) Grossphotography of longitudinal section of the uterus. Placenta (black arrow)attached to extreme thinned uterine wall (white arrow). b,c) histology examinationof uterine wall chorionic villi and trophoblast invasion with fibrin depositwith extreme thinned myometrium with fibrosis demonstrated on hematoxylin andeosin (40× magnification)b) and masson trichrome (200× magnification) c). d) vasculitisin the myometrium (100× magnification). e) a thin layer of the uterus at placentafree site (20× magnification).

## Data Availability

All data analyzed during this study are included in this report.

## Abbreviations

SLE: Systemic lupus erythematosus
PAS: Placenta accreta spectrum
CS: Cesarean section
HE: Hematoxylin and eosin
