# Supplementary material for: Diffuse myometrium thinning and placenta accreta spectrum in a patient with systemic lupus erythematosus (SLE): a case report and review of the literature
Source: BMC Pregnancy Childbirth. 2022 Jul 2;22:535. doi: 10.1186/s12884-022-04864-z (PMC9250175; doi:10.1186/s12884-022-04864-z)
Supplement: Supplementary file 1 — Additional file 1: Figure a) Grossphotography of longitudinal section of the uterus. Placenta (black arrow)attached to extreme thinned uterine wall (white arrow). b,c) histology examinationof uterine wall chorionic villi and trophoblast invasion with fibrin depositwith extreme thinned myometrium with fibrosis demonstrated on hematoxylin andeosin (40× magnification)b) and masson trichrome (200× magnification) c). d) vasculitisin the myometrium (100× magnification). e) a thin layer of the uterus at placentafree site (20× magnification). [file 12884_2022_4864_MOESM1_ESM.docx]

**[Supplementary information]**


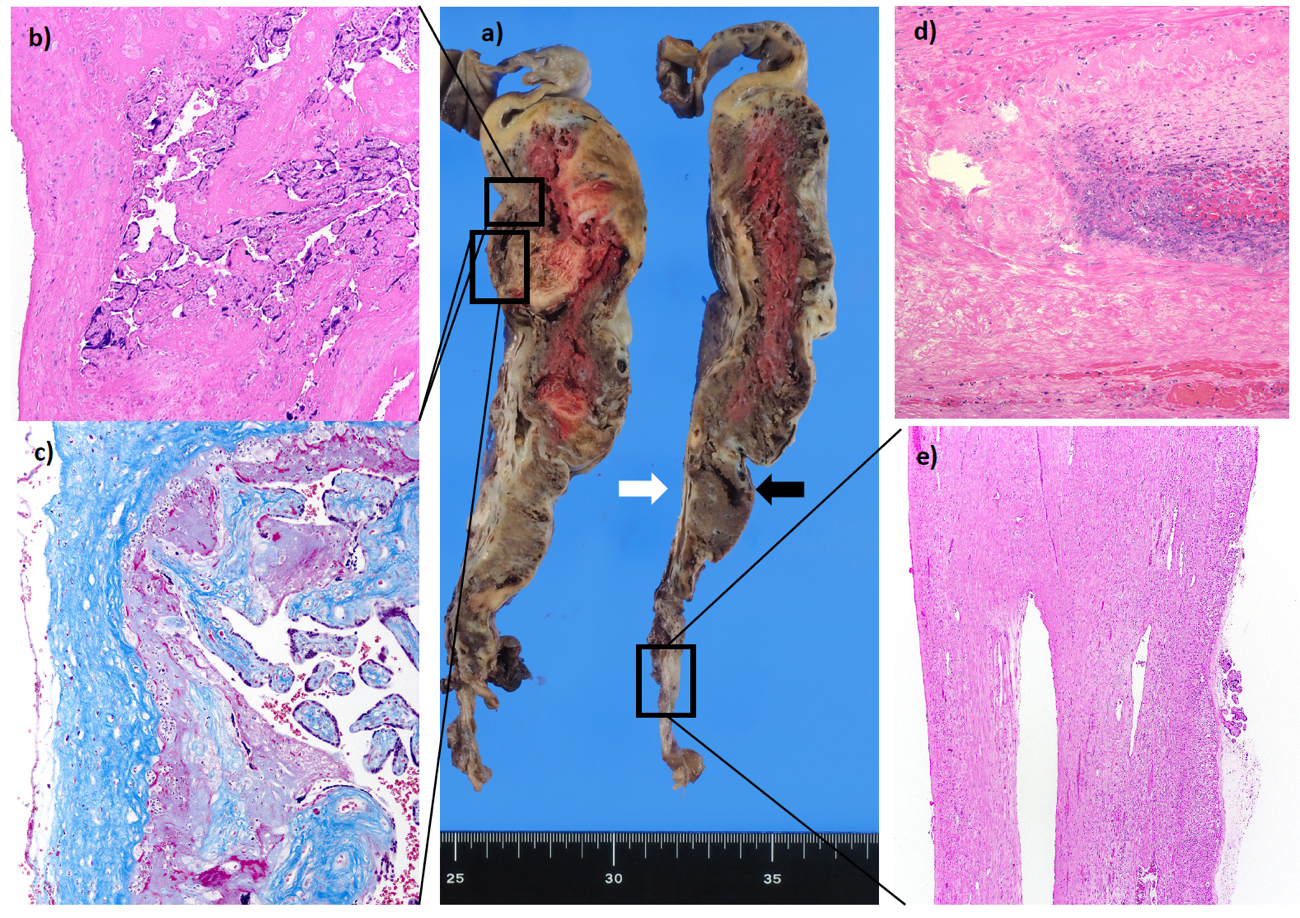


**Figure** a) Gross photography of longitudinal section of the uterus. Placenta (black arrow) attached to extreme thinned uterine wall (white arrow). b,c) histology examination of uterine wall chorionic villi and trophoblast invasion with fibrin deposit with extreme thinned myometrium with fibrosis demonstrated on hematoxylin and eosin (40× magnification)b) and masson trichrome (200× magnification)c). d) vasculitis in the myometrium (100× magnification). e) a thin layer of the uterus at placenta free site (20× magnification).
